# Forecasting induced seismicity in Oklahoma using machine learning methods

**DOI:** 10.1038/s41598-022-13435-3

**Published:** 2022-06-04

**Authors:** Yan Qin, Ting Chen, Xiaofei Ma, Xiaowei Chen

**Affiliations:** 1 Geophysics Group, Earth and Environmental Sciences Division, Los Alamos National Laboratory, Los Alamos, 87545 USA; 2 School of Geosciences, University of Oklahoma, Norman, 73069 USA

**Keywords:** Seismology, Geophysics

## Abstract

Oklahoma earthquakes in the past decade have been mostly associated with wastewater injection. Here we use a machine learning technique—the Random Forest to forecast induced seismicity rate in Oklahoma based on injection-related parameters. We split the data into training (2011.01–2015.05) and test (2015.06–2020.12) periods. The model forecasts seismicity rate during the test period based on input features, including operational parameters (injection rate and pressure), geological information (depth to basement), and modeled pore pressure and poroelastic stress. The results show overall good match with observed seismicity rate (adjusted $R^2$ of 0.75). The model shows that pore pressure rate and poroelastic stressing rates are the two most important features in forecasting. The absolute values of pore pressure and poroelastic stress, and the injection rate itself, are less important than the stressing rates. These findings further emphasize that temporal changes of stressing rates would lead to significant changes in seismicity rates.

## Introduction

The sharp seismicity increase in Oklahoma during the last decade has been associated with wastewater disposal^1–3^. The proposed mechanisms of induced earthquakes include pore pressure diffusion^2,4,5^, poroelastic stress disturbance^6–8^, earthquake interaction effect^6,9–11^, and aseismic slip^12^. Many studies have made seismicity rate forecasts based on known mechanisms. For example, a numerical model that integrates fluid pressurization from injection with a rate-and-state friction model of the earthquake nucleation process is developed to forecast rates of induced seismicity^13^. The study finds that the models with injection data outperform a standard statistical model that only uses prior earthquake observations to forecast induced earthquake activity. A hybrid physical-statistical model combining hydrogeologic modeling and modified Gutenberg-Richter relation is used to forecast seismic hazards^5^. The study finds that seismicity in Oklahoma and Kansas was driven by the rate of injection-induced pressure increases. Another study combines poroelastic modeling and a rate-and-state earthquake nucleation model to forecast the timing and magnitude of induced seismicity^8^. The authors find that while pore-pressure diffusion controls the induced earthquakes in Oklahoma, its impact is significantly enhanced by poroelastic stressing rate changes.

The modeled pore pressure and poroelastic stress are typically calculated based on injection rates, injection locations (including depth), and assumed hydrology models. While injection rate is one of the most important parameters, other operational and geological parameters are also found to be important. For example, the injection depth relative to the crystalline basement strongly correlates with seismic moment release, and the joint effects of injection depth and volume are critical, as injection rate becomes more influential near the basement interface from an advanced Bayesian network^14^. Study shows that most earthquakes in Oklahoma are located where the crystalline basement is likely composed of fractured intrusive or metamorphic rock^15^. A 2-D Pg wave tomography shows that most moderate-size ($\hbox {M}>4$) earthquakes occurred either close to the boundaries between high- and low-seismic velocity zones or within the high-velocity zones^16^. The parameters that are best correlated with earthquakes change with different scales of studies^4^ and different methods^17^.

**Figure 1:**
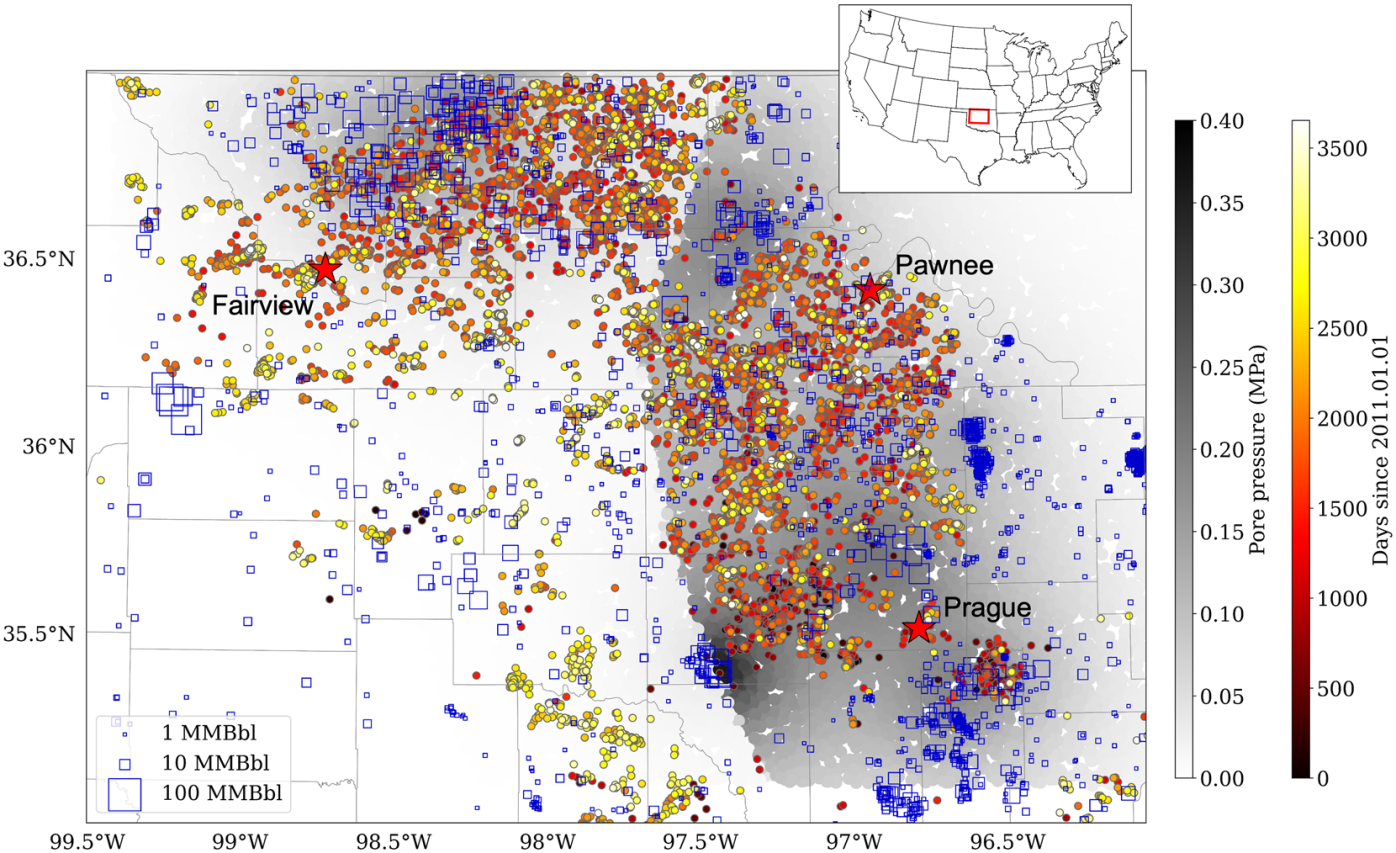
Map view of the declustered seismicity and well injections. Squares: injection wells scaled by the cumulative injection volume from 2011 to 2019. Circles: $\hbox {M}\ge 2.2$ earthquakes from 2011 to 2020, colored by their origin times. The gray lines are county boundaries in Oklahoma. The background shows the modeled pore pressure in June, 2015^5^. The three red stars show the location of $\hbox {M}>5$ earthquakes in Fairview, Pawnee, and Prague in Oklahoma. The inset figure shows the location of our study area (the map is created in Python (Version 3.6); software available at https://www.python.org).

In this study, we compile all the available geological and operational parameters from wastewater injection (Fig. 1) and use a machine learning approach—the Random Forest regression model to forecast the seismicity rate. Without any prior knowledge of the weight of the input features, the model can identify the most important features and improve the understanding of the triggering mechanisms of induced seismicity.

## Results

### Seismicity rate forecast

We build and select injection- and physics-based features at different spatial scales (grid size from $0.6^{\circ }$ to $2.0^{\circ }$ with an increment of $0.1^{\circ }$) using the procedure described in “Methods” section. The features are then used to forecast the seismicity rate in various time windows (30, 60, 90, and 180 days) using Random Forest (RF). To account for the associations of injection and earthquakes at the border of a grid, we use a different grid size to search for earthquakes, that is grid plus ($-\,0.3^{\circ }$ to $0.3^{\circ }$ with an increment of $0.1^{\circ }$ relative to the grid size for features).

We split the data into training (2011.01–2015.05) and test (2015.06–2020.12) dataset. The number of training and test data points is 1696 and 2144, respectively, where each data point consists of a set of injection features and the corresponding seismicity rate in a specific time window. We run the RF forecast for each combination of grid sizes, grid plus, and time windows. We use adjusted $R^2$ (defined in “Methods” section, range from negative to 1) to evaluate the model performance, with high adjusted $R^2$ implying good fit between forecast and observations. The adjusted $R^2$ for training data increase with grid size, while the adjusted $R^2$ for test data first increases and then decrease with grid size. In time, neither training data nor test data show significant difference for different time windows (Fig. S1 in the Supporting Information).

**Figure 2:**
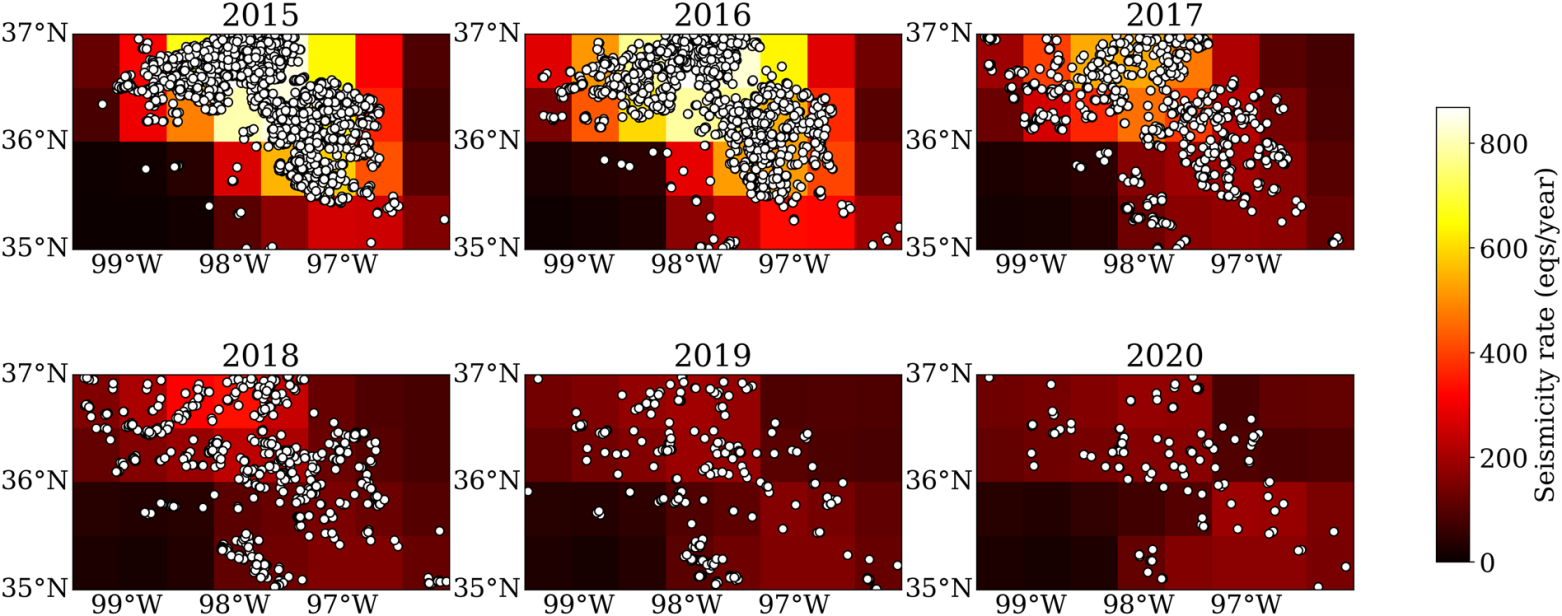
The forecast annual earthquake number for the study area. The white circles denote earthquakes occurred during each year. The annual forecast is the sum of the monthly forecast in each year.

Here, we show the best results of all the models, where the grid size is $1.6^{\circ }$, the time window is 30 days, and the grid plus is $-\,0.2^{\circ }$, and the model results in an adjusted $R^2$ of 0.95 and 0.75 for training and test dataset, respectively. The model forecasts the number of earthquakes in the time window of 30 days. In each grid, we sum the number of earthquakes in 12 months of each year and get the annual seismicity rate. The map view of annual forecasting results is shown in Fig. 2. Consistent with the observations, the seismicity is mainly distributed in central and western Oklahoma, and the seismicity rate remains elevated until 2020 especially for western Oklahoma (300/year $\gg$ 19/year in 2008). The drastic color change between 2016 and 2017 suggests that the model captures the rapid decrease of seismicity in those two years. The annual seismicity rate continues to decrease from 2018 to 2020.

**Figure 3:**
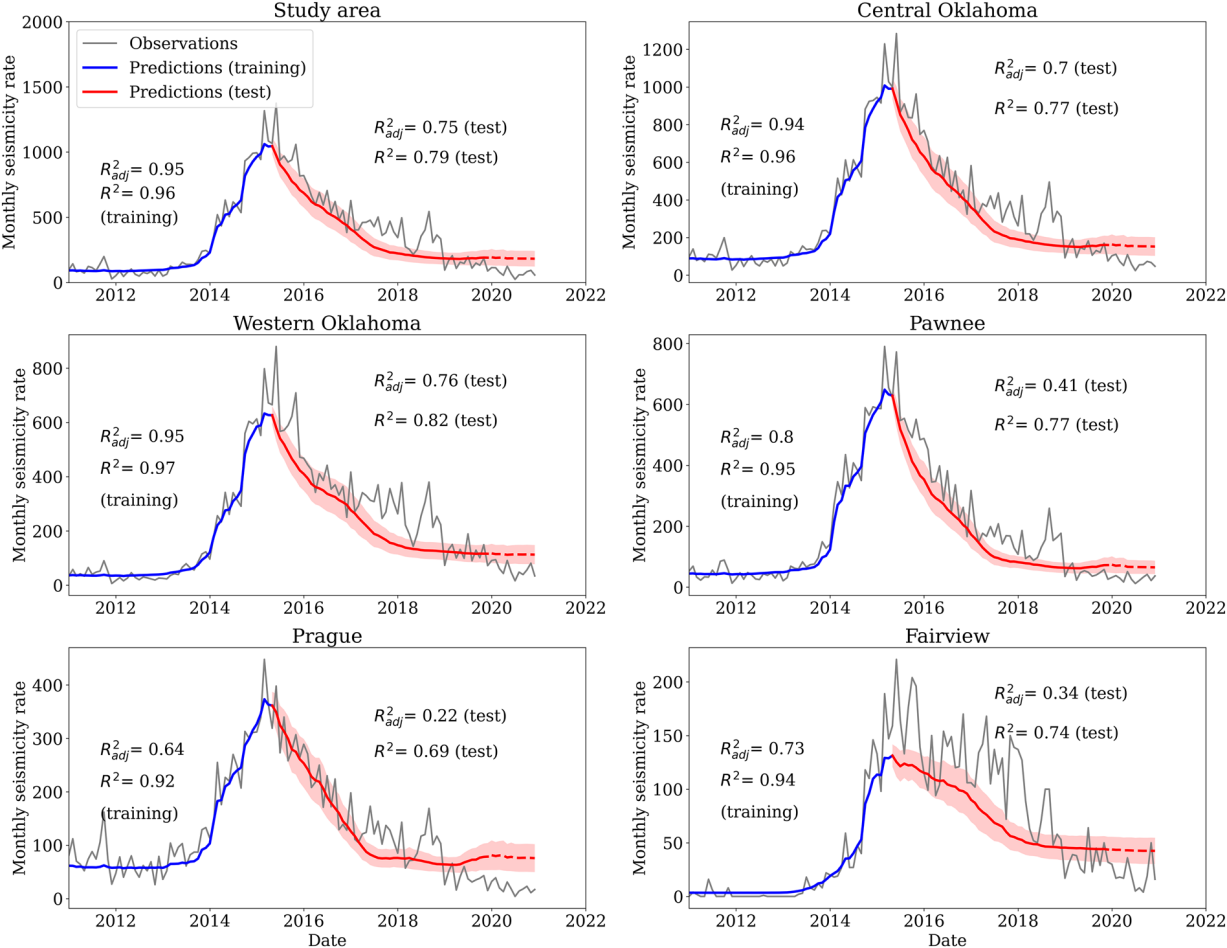
Seismicity rate forecast for different regions, including the whole study area, central Oklahoma (longitude $\ge -98^{\circ }$), western Oklahoma (longitude $<-98^{\circ }$), Pawnee area, Prague area, and Fairview area. The gray lines are observations, and the blue and red lines are forecast results from training and test dataset, respectively. The red dashed line shows forecast for year 2020, where the injection data are not available yet. Red areas indicate one standard deviation from 100 runs. The location of the Pawnee, Prague, and Fairview is shown in Fig. 1. The $R^2$ and $R_{adj}^2$ for training data and test data are listed on each subplot. Notice that $R_{adj}^2$ is smaller than $R^2$, especially for Pawnee, Prague, and Fairview, where the number of data points is small.

In time, we sum the number of earthquakes of selected grids together and plot the seismicity curve for different regions (Fig. 3). Overall, the model forecasts the rapid decreasing seismicity after 2015 with an adjusted $R^2$ of 0.75 for the whole study area. For individual grids (e.g., Pawnee, Prague, and Fairview), the temporal forecast is also reasonable, but the adjusted $R^2$ is lower because of small number of data points in small areas. The results suggest that our model can forecast the first-order seismicity rate for the study area and also reflect heterogeneous relationship between injection and seismicity at different locations. We notice that starting from 2019, the model shows over-prediction for almost the whole study area. The over-prediction likely results from the assumptions about injections, for example, pore pressure data assume constant injection after March, 2018^5^. We also note that the $R^2$ score of training data is higher than that of test data. On one hand, even though we use cross validation in the training process, the model can be over-fitting due to the small dataset. On the other hand, the seismicity rate is not stationary^18^, which makes the forecast process more complicated.

### Feature importance

**Figure 4:**
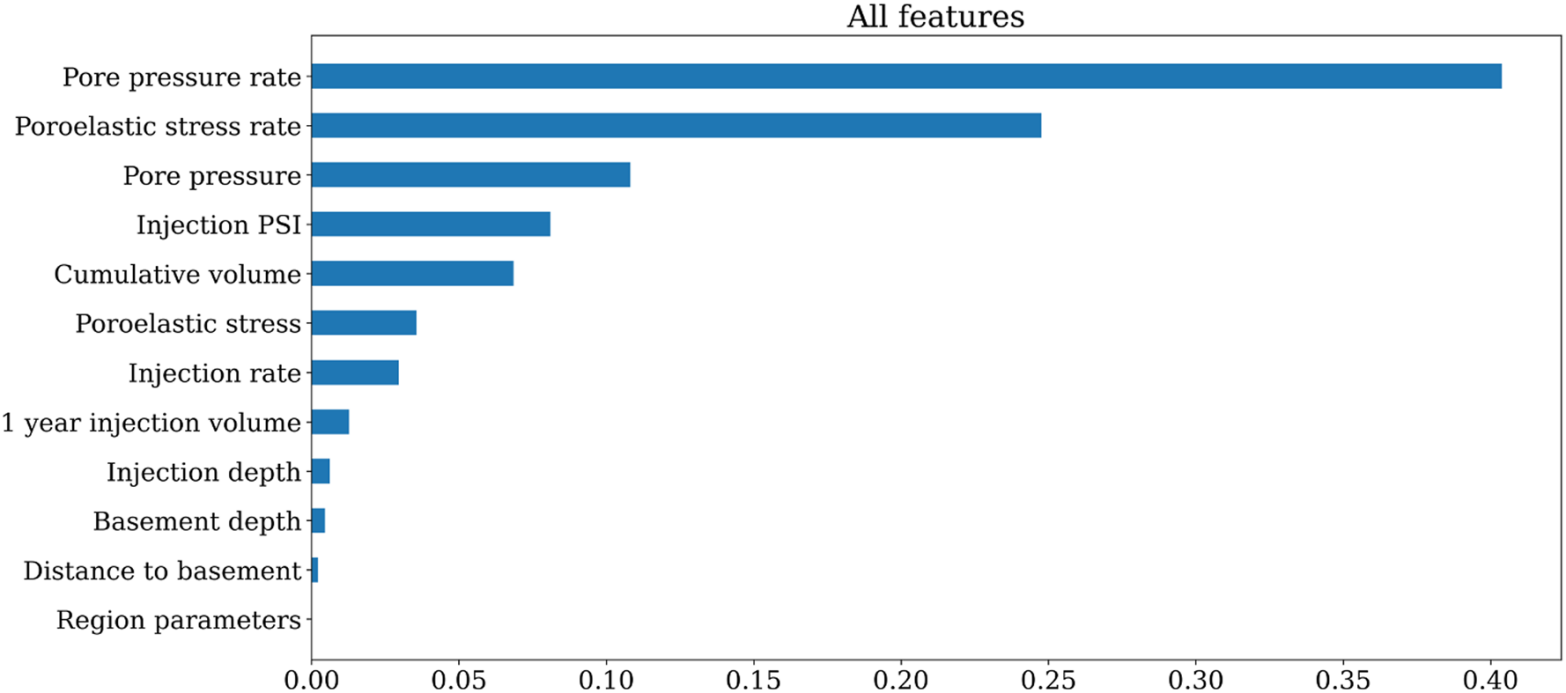
Histogram of feature importance from the RF model. The most important features are pore pressure rate, poroelastic stress rate, and pore pressure.

Figure 4 shows the rank of feature importance from the model. The most important features are pore pressure rate (0.4), poroelastic stress rate (0.25), pore pressure (0.1), injection pressure (0.08), and cumulative volume (0.06). The pore pressure rate and poroelastic stress rate correspond to the two main mechanisms of induced seismicity. The relative importance of the two factors changes slightly with different gridding parameters. The physics-based parameters alone could make forecast as good as those using all the parameters (Figs. S2 and S3 in the Supporting Information).

Besides the above features, injection rate, and 1 year injection volume, and injection depth also show positive correlations to seismicity rate. Without incorporation of pore pressure and poroelastic stress, operational parameters alone can not forecast the seismicity rate well ($R_{adj}^2= 0.2$, Fig. S4). Contrary to the heterogeneity we observed before, the region parameter encoded from the grid number has low feature importance ($<0.001$). Possible explanations are that for physics-based features, hydrogeologic modeling already takes the spatial heterogeneity into account. We add the region parameters with columns of sparse ones and zeros, which could be less informative than the other features. We speculate that adding more meaningful parameters, for example, seismogenic index^5^, geological boundaries^15^, or geospatial correction^14^, could potentially further improve the results.

## Discussion

### Comparison of different forecast models

**Figure 5:**
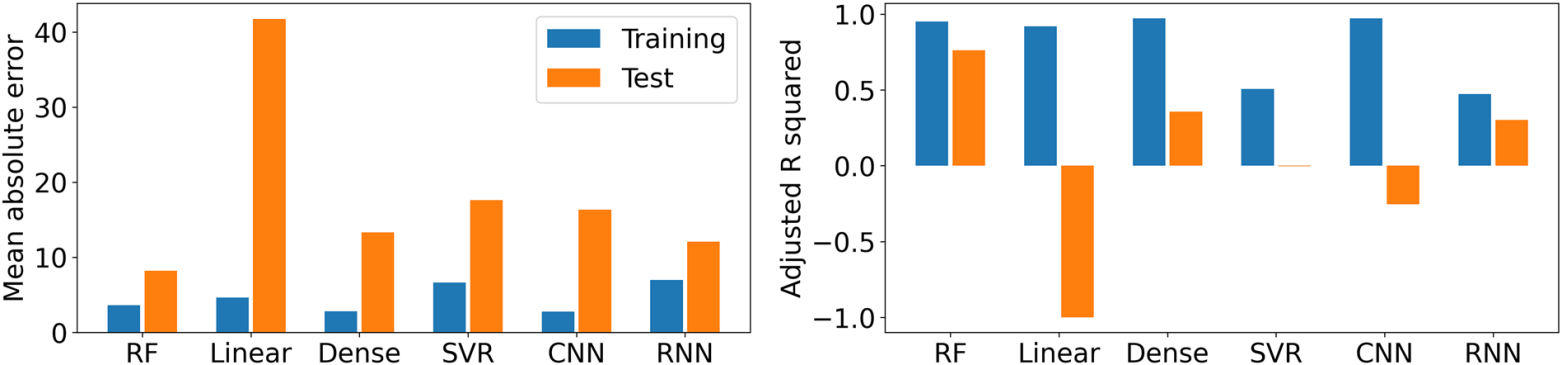
Comparison of model performance. The left panel shows the mean absolute error (MAE), and the right panel shows the adjusted $R^2$. *RF:* Random Forest, *Linear:* least square Linear Regression, *Dense:* sequential model of three linear regression layers stacked, *SVR:* Support Vector Regression model, *CNN:* sequential model with a 1D Convolutional layer, *RNN:* sequential model with a long short-term memory layer.

For comparison, we use different machine learning models to forecast seismicity based on the same set of training and test data. The models include a least square Linear Regression model (referred to as Linear model), a sequential model with three Dense layers stacked (referred to as Dense model) where each Dense layer includes linear regression and activation functions, a Support Vector Regression model (referred to as SVR model), a sequential model with a 1D Convolutional layer and a Dense layer (referred to as CNN model), and a sequential model with a Long Short-Term Memory (LSTM) layer and a Dense layer (referred to as RNN model). Different from the Linear model, the Support Vector Regression is a supervised learning algorithm to predict discrete values by finding the best-fit hyperplane with the maximum number of points. The algorithm acknowledges the non-linearity in the data and is robust to outliers. Convolutional layers apply a convolution operation to the input and pass the results to the next layer, which are used to extract features from the input data and have been used widely in earthquake detection^19^. A LSTM layer learns long-term dependencies between time steps in time series and sequence data, which has also been used in earthquake detection^20^. We use the aforementioned adjusted $R^2$ and mean absolute error (MAE) as metrics to compare the performance of different models. MAE is defined as,

1 $$\begin{aligned} MAE =\frac{\sum _{i=1}^{n} |f_i-y_i|}{n} \end{aligned}$$

where $y_i$ is the observed seismicity rate, $f_i$ is the corresponding prediction of $y_i$. The results are shown in Fig. 5.

The models are ranked from high to low by their performance on test data: RF, Dense, RNN, SVR, CNN, and the Linear model. Random Forest outperforms the other models. Besides Random Forest model, considering our training dataset is relatively small, the Dense model is a good balance between model complexity and fit of data. RNN can keep track of arbitrary long-term dependencies in the input sequences, which might help with some complex situations in earthquake forecast, e.g., different time lags between injection and earthquake occurrence.

We also check against the forecast results from two physics-based forecasting methods^8,21^. Since the study area and forecast target are different, it is not easy to make one-to-one comparison. We qualitatively compare our model to the physics-based forecasting methods. Both methods can forecast the decreasing seismicity in recent years. Our model can make forecast at different time windows (from 30 to 180 days) and has better resolution than the yearly forecast from physics-based models. In terms of spatial resolution, the physics-based models are better, as the performance of our model decreases if the grid size is smaller. Our model could also identify the triggering mechanisms independently from a set of input features, which suggests that the application of machine learning methods could be used to study the physics behind observations. On the other hand, we have seen model improvements by adding physics-based parameters, e.g., pore pressure and poroelastic stress. The performance of seismic forecasting may be further improved using physics-informed machine learning models.

### Physical mechanism of induced seismicity

The feature importance analysis suggests that physics-based parameters are more important than operational and geological parameters, because the modeling of pore pressure and poroelastic stress incorporates the injection rate, injection depth, and other hydrogeologic parameters. These results are consistent with the previously known mechanism for induced seismicity that the increase of pore pressure reduces effective normal stress and promotes fault failure. The causal relationship between pore pressure diffusion and seismicity has been examined by both observations^4,22,23^ and modeling^2,5^. The effect of poroelastic stress on induced seismicity becomes more significant at relatively far distance^6–8^.

Among the physical parameters, the pore pressure rate and poroelastic stressing rate are the two most important parameters, with much higher ranking than absolute values of pore pressure and poroelastic stress. Studies^8,24^ have shown good agreement between observation and forecast seismicity rate using stressing rates based on rate-and-state model^25^. Their results suggested that variable injection rates that caused large stressing rate changes led to higher seismicity rate. Our machine learning analysis obtains consistent conclusion (stressing rate is more important) and similar forecasting performances. These results suggest promising applications of machine learning techniques in extracting the physical mechanism of induced seismicity and forecasting induced seismicity rates.

Besides the identified physical features, the various relationship between input features and forecast results can statistically provide information about the triggering mechanisms. For example, the parameter grid plus is used to account for the correlations between earthquakes and injection at grid borders. Results show the adjusted $R^2$ varies for different grid plus sizes from $-\,0.3^{\circ }$ to $0.3^{\circ }$. Grid plus of $-\,0.1^{\circ }$ and $-\,0.2^{\circ }$ generates the best results by enclosing the earthquakes at the grid borders with a larger grid of injection wells. The $R^2_{adj}$ starts to decrease if the grid plus is too large (e.g., $0.2^{\circ }$ and $0.3^{\circ }$), which suggests that at a larger distance (around 20–30 km), the wells should not be associated with the earthquakes within the grid. These findings are consistent with the spatial range of pore pressure diffusion influence from previous studies^2,3^.

In this paper, we compile the injection-related parameters in Oklahoma and use them to forecast seismicity rate in a Random Forest regression model. The model can forecast the first-order seismicity rate in Oklahoma. The model also identifies pore pressure rate and poroelastic stress rate as the most important features to forecast induced seismicity. The results demonstrate potential to use machine learning methods to forecast seismicity and to understand the physics behind the induced seismicity.

## Methods

### Earthquake data

We use the earthquake catalog from January 2011 to December 2020 from the Oklahoma Geological Survey (OGS)^26^. The catalog has a completeness of magnitude ($M_c$) of 2.2 based on maximum curvature of the frequency-magnitude distribution^27^ (Fig. S5 in the Supporting Information). Studies have shown evidence of earthquake interactions among clustered events in Oklahoma^6,9–11^. Since we do not account for earthquake interactions, we decluster the catalog by removing the aftershocks following the recently revised spatial and temporal windows proposed for Oklahoma earthquakes^28^. Figure 1 shows the declustered catalog with a magnitude cutoff of 2.2.

### Wastewater injection and geological parameters

We obtain the monthly injection data from 2011 to 2019 from the Oklahoma Corporation Commission (OCC). We select wells with the cumulative injection volume since 2005 larger than 1000 bbl (Fig. 1). For each well, the spikes in injection volume larger than ten times of the average values are removed to avoid possibly wrong records. We also extract the basement depth at each injection location^29^ and calculate the distance from the injection depth to the basement. The final parameters include monthly injection rate, yearly (the most recent 1 year) and cumulative injection volume, injection pressure (PSI), injection depth, basement depth, and distance from the injection depth to the basement. Since the injection data are not available for 2020 and 2021 at the time of the study, we assume the injection parameters stay constant after the last month in 2019.

### Pore pressure and poroelastic stress data

To include physics-based features from injections, we add pore pressure data (Fig. 1) and poroelastic stress data from hydrogeologic modeling. The pore pressure data^5^ are calculated for each month at 25,000 randomly selected points in Oklahoma and have been used in a seismogenic-index based model to successfully forecast statewide seismic hazards. The poroelastic data^8^ are computed at gridded points in central and western Oklahoma by assuming an optimal NW and NE fault orientation. The data have been used in seismicity forecast in a rate-and-state model^8^. We should mention that the pore pressure data and poroelastic stress data are derived from different modeling parameters and different assumptions about the injection rate. The former^5^ assumes the injection stays constant after March 2018, and the latter^8^ hypothesizes an injection shut-in after April 2017. Fortunately, we use the tree-based model Random Forest which does not directly compare the absolute values of pore pressure and poroelastic stress, so we can treat them as two independent features.

### Feature selection and forecast target

We build a list of features from injection and physical parameters to forecast seismicity rate. First, the study area (inset figure in Fig. 1) is divided into uniform grids ($0.6^{\circ }$ to $2.0^{\circ }$ with an increment of $0.1^{\circ }$), and in each grid we search for injection wells, modeled pore pressure and poroelastic stress data points for each month. The statistics, including sum, mean, maximum, minimum, different percentiles of the injection parameters (rate and pressure), geological parameters (depth), pore pressures, and poroelastic stress data in each grid in each month form the initial features. Then we remove the redundant features that are highly correlated with each other and the features that do not show good correlation with the target (cross-correlation coefficient $<0.35$). Next, to account for possible delayed response between input features and the target, we add history data (the feature difference between the current month and the previous months, Fig. S6 in the Supporting Information) to the feature list. Finally, we add a region number parameter (encoded to multiple columns of zeros and ones) to represent the geological features. Table 1 lists the selected features.

The forecast target is the earthquake number in the next time window (30, 60, 90, and 180 days). Considering that at the borders, some earthquakes might be associated with the injection wells outside the grid, we select a different grid size (referred to as grid plus, from − 0.3$^{\circ }$ to 0.3$^{\circ }$, Fig. S7 in the Supporting Information) to search for earthquakes. A negative grid plus means that the grid for earthquake search is smaller than that in well search, and vice versa. We also apply a moving window of one-fourth of the grid size to obtain more data points for spatial variations (Fig. S8 in the Supporting Information).

**Table 1:** The list of selected input features for Random Forest forecast.

Pore pressure rate*	Pore pressure rate (mean), pore pressure rate (max), pore pressure rate (variance), pore pressure rate (20th percentile), pore pressure rate (gradient between minimum and maximum values in space)
Pore pressure*	Pore pressure (mean), pore pressure (max)
Poroelastic stress rate (PES rate)*	PES rate (mean), PES rate (max), PES rate (variance), PES rate (20th percentile), PES rate (gradient between minimum and maximum values in space)
Poroelastic stress (PES)*	PES (mean)
Injection rate*	Monthly injection volume (sum)
Injection PSI*	Monthly injection pressure (skewness)
Cumulative volume*	Cumulative injection volume (20th percentile)
1 year injection volume*	1 year injection volume (max), 1 year injection volume (40th percentile)
Injection depth	Well depth (mean), well depth (max), well depth (min), well depth (variance)
Basement depth	Basement depth (mean), basement depth (max), basement depth (min), basement depth (variance)
Distance to basement	Distance (mean), distance (max), distance (min), distance (variance)
Region parameters	Grid number 0–31 to represent different locations

The complete input features also include the history data of the features denoted by asterisk (*).

### Random Forest model

We use a machine learning technique—Random Forest (RF) to forecast the seismicity rate. The RF model makes predictions of the target variable (seismicity rate) based on a list of input features^30–32^ (Table 1). We split the data into training (2011.01–2015.05) and test (2015.06–2020.12) dataset. The training dataset covers the seismicity onset and peak, and the test dataset starts about 1 month following the peak. We use grid search with 3-fold validations to optimize the hyper-parameters of the RF model. The best-fit model has the hyper-parameters as follows, tree number of 20, maximum depth of 5, the minimum number of samples to split of 2, and the minimum number of samples for a leaf node of 4. The model is then used to make forecast for the test data. A metric of adjusted $R^2$ score ($R^2_{adj}$) is selected to measure how well the model predictions approximate the observations. The $R^2_{adj}$ is defined as follows,

2 $$\begin{aligned} R^2_{adj} =1-\frac{(1-R^2)(n-1)}{(n-k-1)} \end{aligned}$$

where *n* is the total sample size, *k* is the number of independent features, and $R^2$ is defined as,

3 $$\begin{aligned} R^2 =1-\frac{\sum _{i} (f_i-\bar{y})^2 }{ \sum _{i} (y_i-\bar{y})^2} \end{aligned}$$

where $\bar{y}$ is the mean of the observed seismicity rate $y_i$, $f_i$ is the corresponding prediction of $y_i$. The $R^2$ also represents how well the predictions fit the observation data and tends to increase with the number of features. The adjusted $R^2$ compensates for the addition of variables and only increases if the new variable enhances the model above what would be obtained by probability. So the adjusted $R^2$ is used to prevent data over-fitting and unwarranted high $R^2$ value from including too many features. An $R^2_{adj}$ value equal to one implies perfect prediction of the observation data, and a value less than or equal to 0 indicates a model that has no predictive value.

We also calculate the permutation-based feature importance. This method will randomly shuffle each feature and compute the change in the model’s performance. The features which impact the performance the most are the most important one. The importance values are between 0 and 1, with values close to 1 meaning the highest importance. The rank of the features helps understand the physics behind the induced seismicity. For each category of features, we sum the contributions of its all related statistical features (e.g., sum and mean) together.

## Supplementary Information

Supplementary Figures.

## References

[1] Ellsworth WL (2013). Injection-induced earthquakes. Science.

[2] Keranen KM, Weingarten M, Abers GA, Bekins BA, Ge S (2014). Sharp increase in central Oklahoma seismicity since 2008 induced by massive wastewater injection. Science.

[3] Yeck, W. *et al.* Far-field pressurization likely caused one of the largest injection induced earthquakes by reactivating a large preexisting basement fault structure. *Geophys. Res. Lett.* **43**. 10.1002/2016GL070861 (2016).

[4] Haffener J, Chen X, Murray K (2018). Multiscale analysis of spatiotemporal relationship between injection and seismicity in Oklahoma. J. Geophys. Res. Solid Earth.

[5] Langenbruch C, Weingarten M, Zoback MD (2018). Physics-based forecasting of man-made earthquake hazards in Oklahoma and Kansas. Nat. Commun..

[6] Segall P, Lu S (2015). Injection-induced seismicity: Poroelastic and earthquake nucleation effects. J. Geophys. Res. Solid Earth.

[7] Goebel T, Weingarten M, Chen X, Haffener J, Brodsky E (2017). The 2016 mw5.1 fairview, oklahoma earthquakes: Evidence for long-range poroelastic triggering at> 40 km from fluid disposal wells. Earth Planet. Sci. Lett..

[8] Zhai G, Shirzaei M, Manga M, Chen X (2019). Pore-pressure diffusion, enhanced by poroelastic stresses, controls induced seismicity in Oklahoma. Proc. Natl. Acad. Sci..

[9] Chen X (2017). The Pawnee earthquake as a result of the interplay among injection, faults and foreshocks. Sci. Rep..

[10] Pennington C, Chen X (2017). Coulomb stress interactions during the Mw 5.8 Pawnee sequence. Seismol. Res. Lett..

[11] Qin Y, Chen X, Carpenter BM, Kolawole F (2018). Coulomb stress transfer influences fault reactivation in areas of wastewater injection. Geophys. Res. Lett..

[12] Eyre TS (2019). The role of aseismic slip in hydraulic fracturing-induced seismicity. Sci. Adv..

[13] Norbeck J, Rubinstein JL (2018). Hydromechanical earthquake nucleation model forecasts onset, peak, and falling rates of induced seismicity in Oklahoma and Kansas. Geophys. Res. Lett..

[14] Hincks T, Aspinall W, Cooke R, Gernon T (2018). Oklahoma’s induced seismicity strongly linked to wastewater injection depth. Science.

[15] Shah AK, Keller GR (2017). Geologic influence on induced seismicity: Constraints from potential field data in Oklahoma. Geophys. Res. Lett..

[16] Pei S, Peng Z, Chen X (2018). Locations of injection-induced earthquakes in Oklahoma controlled by crustal structures. J. Geophys. Res. Solid Earth.

[17] Weingarten M, Ge S, Godt JW, Bekins BA, Rubinstein JL (2015). High-rate injection is associated with the increase in us mid-continent seismicity. Science.

[18] Montoya-Noguera S, Wang Y (2017). Bayesian identification of multiple seismic change points and varying seismic rates caused by induced seismicity. Geophys. Res. Lett..

[19] Zhu W, Beroza GC (2019). Phasenet: A deep-neural-network-based seismic arrival-time picking method. Geophys. J. Int..

[20] Mousavi SM, Ellsworth WL, Zhu W, Chuang LY, Beroza GC (2020). Earthquake transformer-an attentive deep-learning model for simultaneous earthquake detection and phase picking. Nat. Commun..

[21] Rubinstein, J. L., Barbour, A. J. & Norbeck, J. H. Forecasting induced earthquake hazard using a hydromechanical earthquake nucleation model. *Seismol. Res. Lett.* (2021).

[22] Langenbruch C, Shapiro SA (2010). Decay rate of fluid-induced seismicity after termination of reservoir stimulations post injection seismicity. Geophysics.

[23] Shapiro SA, Dinske C, Langenbruch C, Wenzel F (2010). Seismogenic index and magnitude probability of earthquakes induced during reservoir fluid stimulations. Leading Edge.

[24] Barbour AJ, Norbeck JH, Rubinstein JL (2017). The effects of varying injection rates in Osage county, Oklahoma, on the 2016 Mw 5.8 Pawnee earthquake. Seismol. Res. Lett..

[25] Dieterich J, Cayol V, Okubo P (2000). The use of earthquake rate changes as a stress meter at Kilauea volcano. Nature.

[26] Walter JI (2020). The Oklahoma geological survey statewide seismic network. Seismol. Res. Lett..

[27] Wiemer S, Wyss M (2000). Minimum magnitude of completeness in earthquake catalogs: Examples from Alaska, the western United States, and Japan. Bull. Seismol. Soc. Am..

[28] Rosson Z, Walter J, Goebel T, Chen X (2019). Narrow spatial aftershock zones for induced earthquake sequences in Oklahoma. Geophys. Res. Lett..

[29] Crain, K. D. & Chang, J. C. Elevation map of the top of the crystalline basement in oklahoma and surrounding states. *Oklahoma Geol. Surv. Open-File Rept. OF1-2018* (2018).

[30] Ho, T. K. Random decision forests. In *Proceedings of 3rd International Conference on Document Analysis and Recognition*, Vol. 1, 278–282 (IEEE, 1995).

[31] Ho TK (1998). The random subspace method for constructing decision forests. IEEE Trans. Pattern Anal. Mach. Intell..

[32] Rouet-Leduc B (2017). Machine learning predicts laboratory earthquakes. Geophys. Res. Lett..

