# Supplementary material for: Forecasting induced seismicity in Oklahoma using machine learning methods
Source: Sci Rep. 2022 Jun 4;12:9319. doi: 10.1038/s41598-022-13435-3 (PMC9167295; doi:10.1038/s41598-022-13435-3)
Supplement: Supplementary file 1 — Supplementary Figures. [file 41598_2022_13435_MOESM1_ESM.pdf]

Supporting Information for "Forecasting Induced Seismicity in Oklahoma using Machine Learning Methods"

Yan Qin[1], Ting Chen[1], Xiaofei Ma[1], Xiaowei Chen[2]
[1]Earth and Environmental Sciences Division – Geophysics Group, Los Alamos National Laboratory, Los Alamos, NM, 87545
[2]School of Geosciences, University of Oklahoma, Norman, OK, 73069

Contents of this file

1. Figures S1 to S8

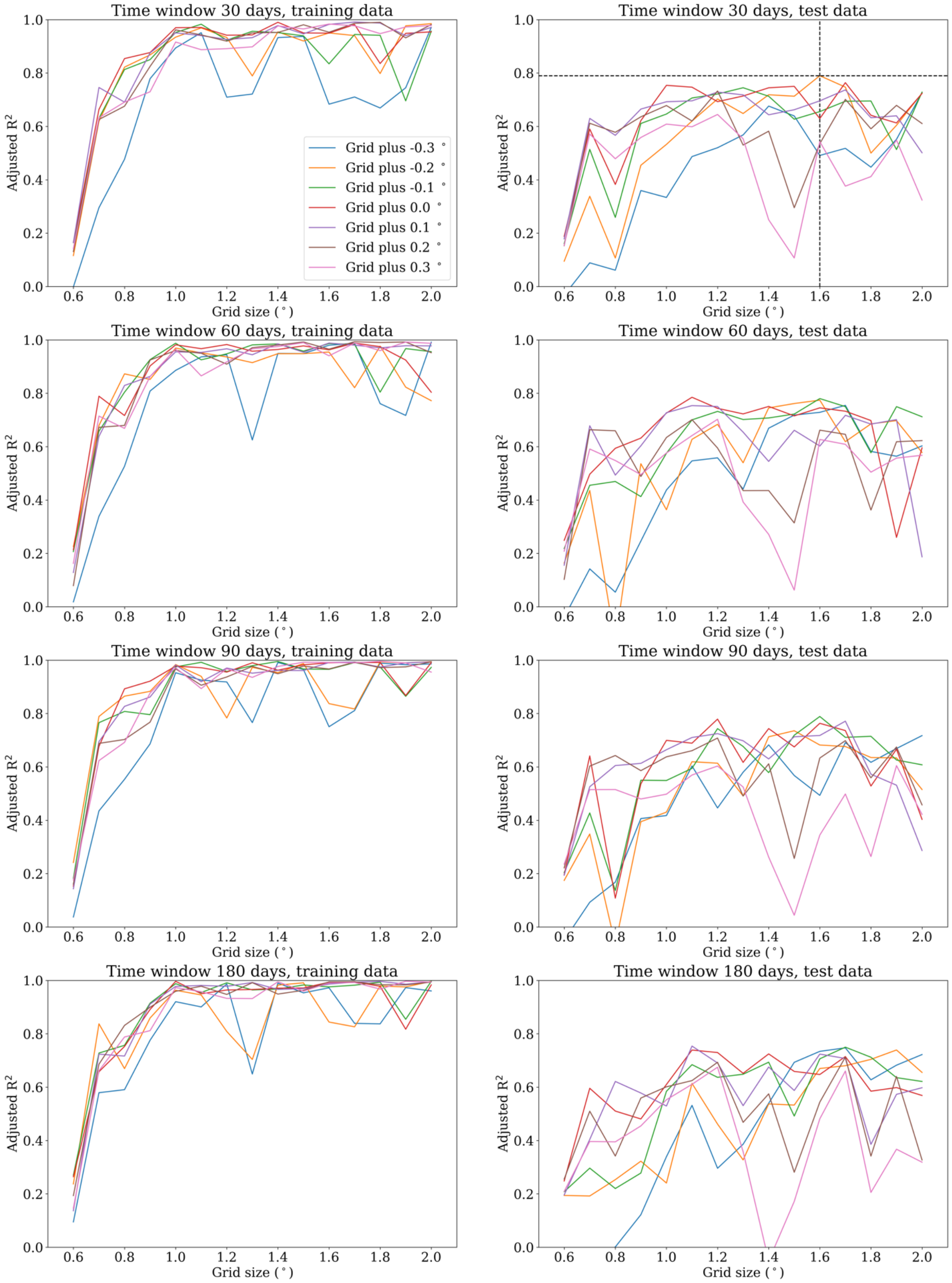

Figure S1. The variation of adjusted R2 for different combination of grid sizes, grid plus, and time windows. The model performance for training data do not show much variations for grid size larger than 1.0˚. The best parameter combination based on test data is denoted by dashed lines, where grid size is 1.6˚, time window is 30 days, and grid plus is -0.2˚.

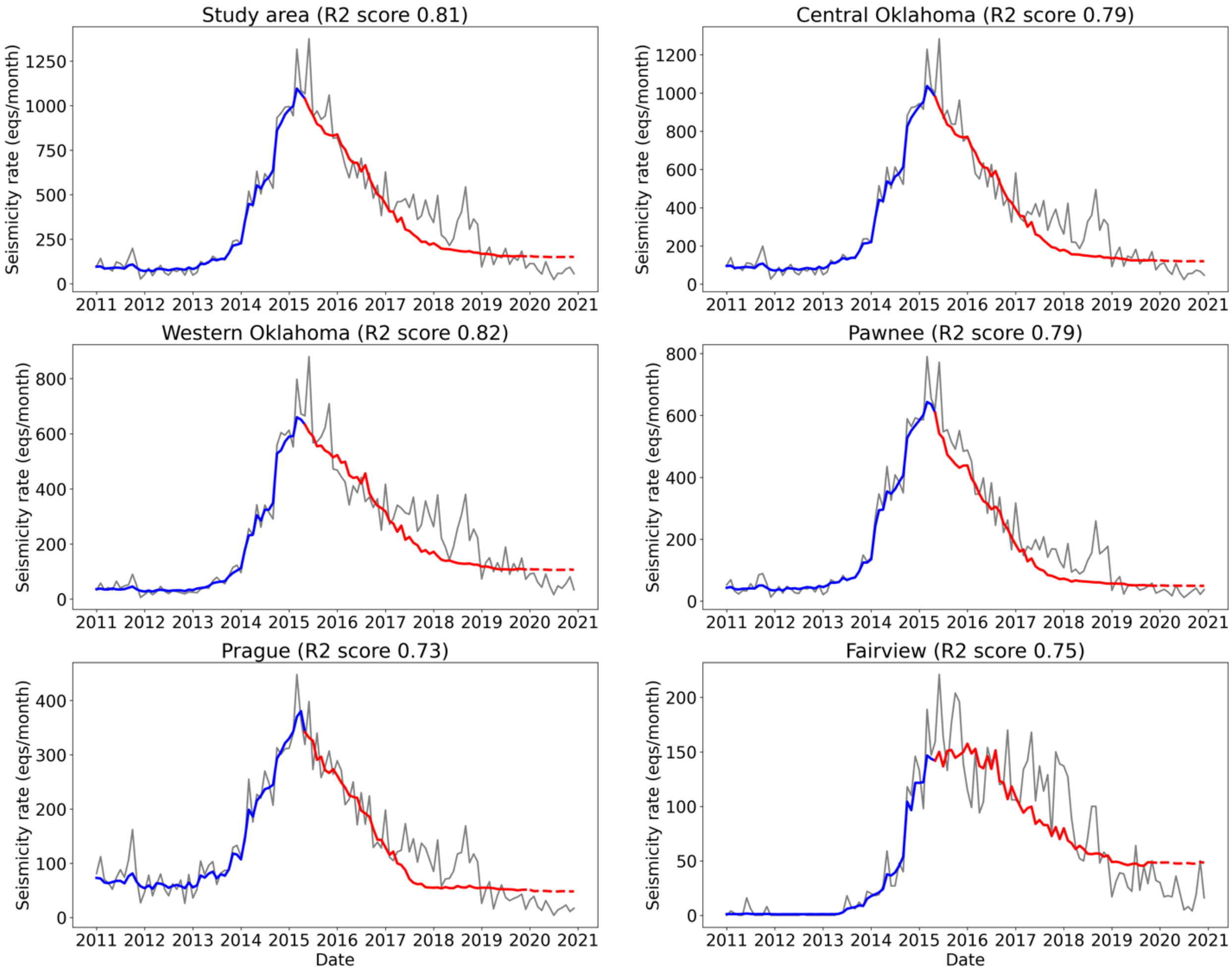

Figure S2. Seismicity rate forecast using only physics-based parameters (pore pressure and poroelastic stress) and region parameters in random forest in (a) the whole study area, (b) central Oklahoma, (c) western Oklahoma, (d) Pawnee sequence, (e) Prague sequence, and (f) Fairview sequence. The gray lines are observations, and the blue and red lines are forecasting results for training and test dataset, respectively. The red dashed line shows forecast for year 2020, where the injection data are not available yet. The R^2 score for test data is shown in the title for each subfigure.

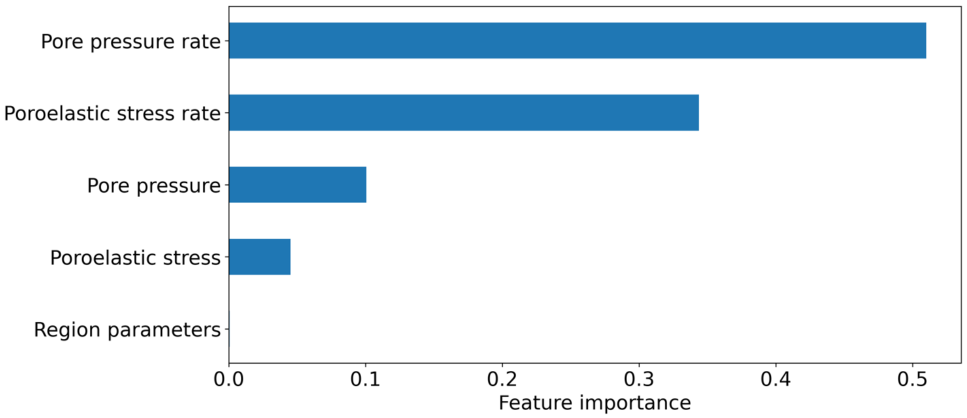

Figure S3. Histogram of feature importance for random forest model using only physics-based parameters and region parameters.

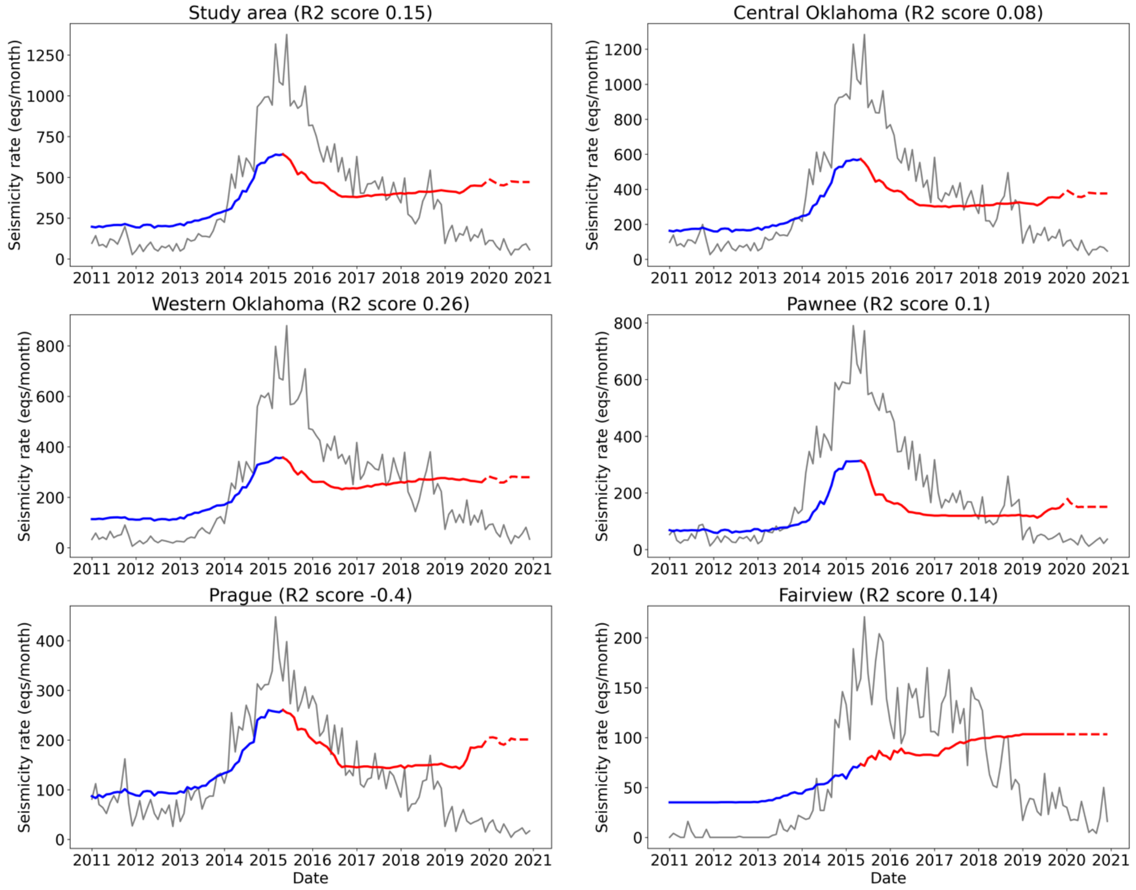

Figure S4. Seismicity rate forecast using only operational parameters and region parameters in random forest in (a) the whole study area, (b) central Oklahoma, (c) western Oklahoma, (d) Pawnee sequence, (e) Prague sequence, and (f) Fairview sequence. The gray lines are observations, and the blue and red lines are forecasting results for training and test dataset, respectively. The red dashed line shows forecast for year 2020, where the injection data are not available yet. The R^2 score is shown in the title for each subfigure.

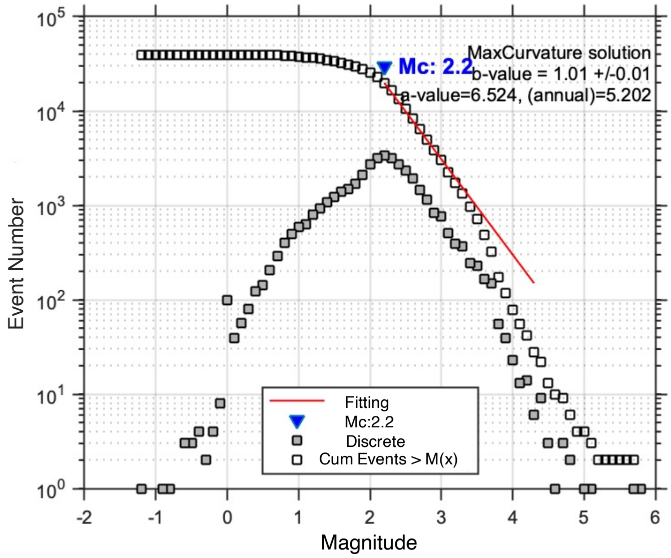

Figure S5. Magnitude distribution of the Oklahoma Geological Survey (OGS) catalog (2011--2020). The completeness of magnitude is 2.2.

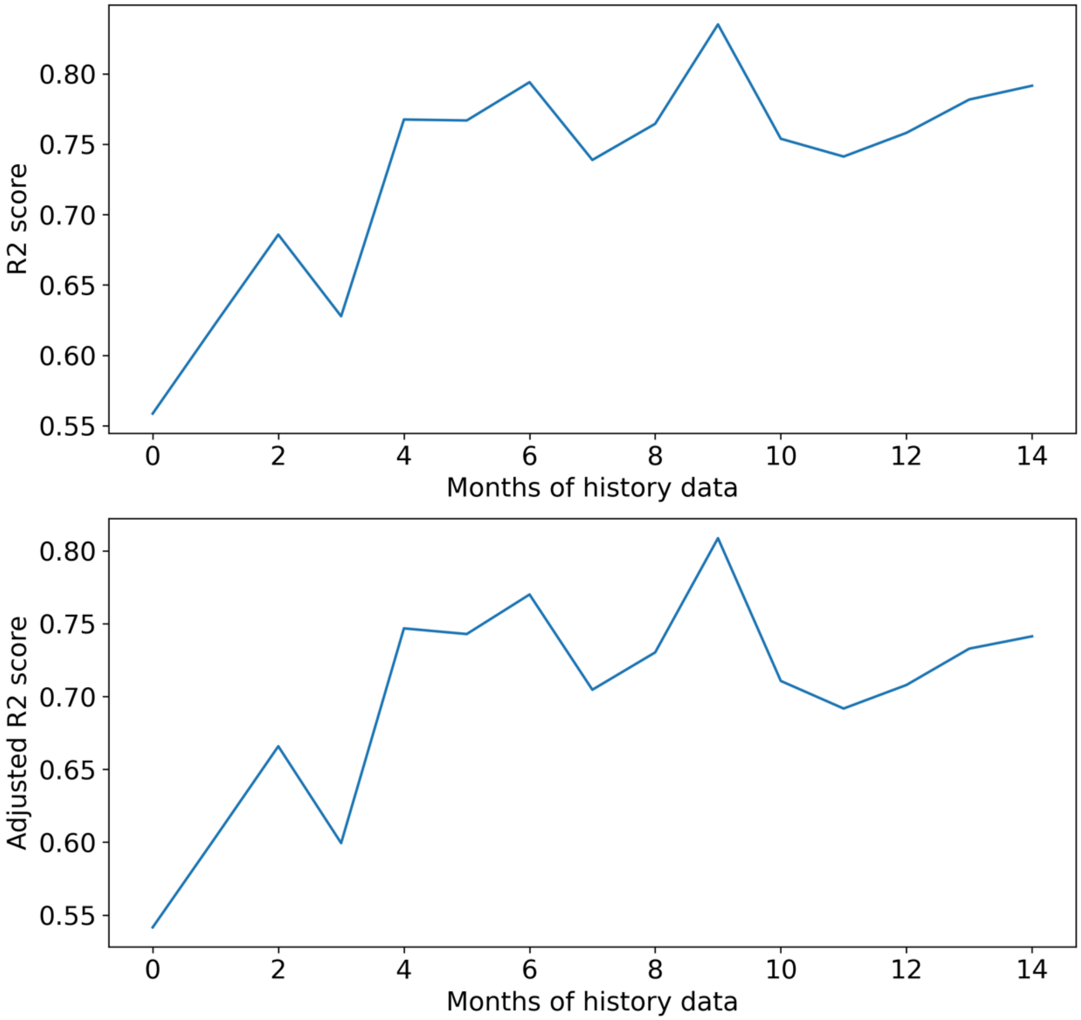

Figure S6. The R^2 score and adjusted R2 score by adding history data to the feature list. Grid size is 1.6˚, and time window is 30 days.

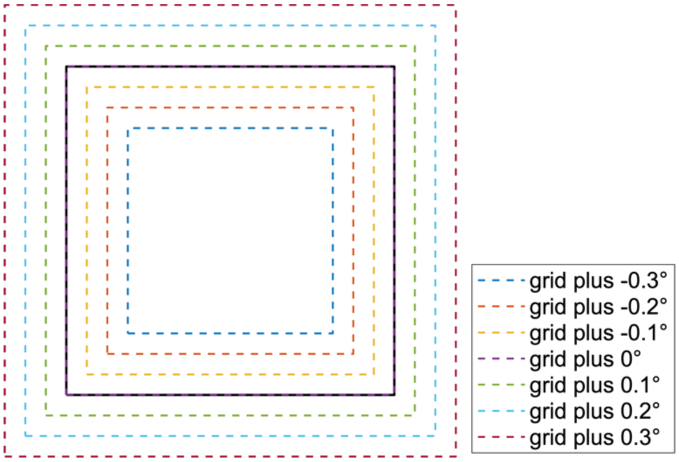

Figure S7. The diagram shows the meaning of grid plus for a grid size of 1.6°. We search for injections, pore pressure, poroelastic stress points in the black grid. We search for earthquakes in the grids of different sizes (dashed lines).

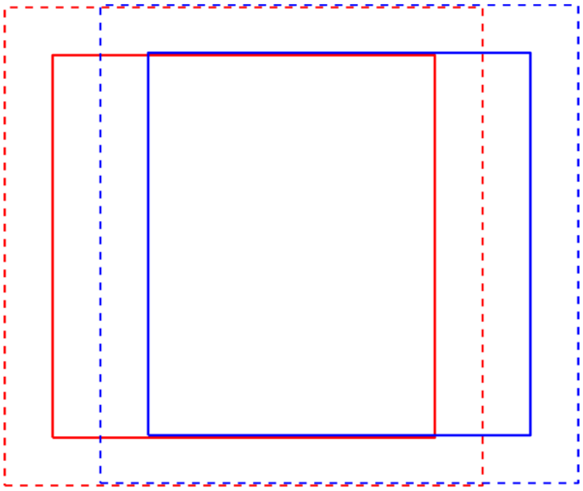

Figure S8. The diagram shows the concept of moving window (grid size 1.6°). The red and blue squares show the current and the next space window. The step between them is one-fourth of the grid size. The solid and dashed squares are for feature search and earthquake search, respectively (Figure S2).

Reference

Zhai, G., Shirzaei, M., Manga, M., & Chen, X. (2019). Pore-pressure diffusion, enhanced by poroelastic stresses, controls induced seismicity in Oklahoma. *Proceedings of the National Academy of Sciences*, *116*(33), 16228-16233.
